# Metaphorical Humor in Satirical News Shows: A Content Analysis

**DOI:** 10.1080/10926488.2022.2160252

**Published:** 2023-05-25

**Authors:** Ellen Droog, Christian Burgers

**Affiliations:** a Vrije Universiteit Amsterdam; b University of Amsterdam

## Abstract

Satirical news is often characterized as a hybrid genre that consists of three important communicative functions: it is (1) humoristic, (2) informative, and (3) evaluative. The Humoristic Metaphors in Satirical News (HMSN) typology demonstrates that metaphors can be utilized by satirists to express this hybridity by consisting of a combination of one or more of satire’s core communicative functions. Nevertheless, the underlying principles through which metaphors are capable of humorously explaining and/or criticizing current affairs are less clear. To broaden our understanding of how metaphorical humor is used in satirical news to fulfill these functions, we integrate the HMSN typology with the General Theory of Verbal Humor (GTVH). The GTVH assumes that all verbal humor must draw from six interdependent Knowledge Resources (KRs). Through a content analysis of metaphorical humor used across various American satirical news shows, we investigated how these KRs are used to fulfill satire’s core communicative functions across the various metaphorical sub-types of the HSMN typology. We found that: (1) some KRs can help fulfill the communicative function(s) of metaphorical jokes, while (2) some KRs constrain the options available for the expression of certain communicative function(s) or other KRs.

Satirical news shows like *Last Week Tonight* (US), the *Heute-Show* (Germany) or *Zondag met Lubach* (the Netherlands), have become increasingly popular across the globe. While it is difficult to present one overarching definition of satire (Condren, 2012), many scholars agree that satirical news has three important communicative functions: it is (1) humoristic, (2) informative, and (3) evaluative (Baym, 2005). Satire fulfills these functions by adopting conventions from the genres of comedy, news and political opinion, and is therefore often characterized as a hybrid genre (Baym, 2005). The hybridity of satirical news is for example reflected in surface features, such as the use of a news anchor behind a desk or over-the-shoulder graphics (Baym, 2005). On a higher discourse level we see that the hybridity of this genre is reflected in the adoption of linguistic register features of both news as well as political fiction (Brugman et al., 2021). Nevertheless, how this hybridity manifests on a lower discourse level has received little scholarly attention so far (Droog et al., 2020). Therefore we need a better understanding of the specific steps satirists make in order to construe this hybrid genre.

To better characterize and understand such lower level discourse features within the genre of satirical news, Droog et al. (2020) introduced the Humoristic Metaphors in Satirical News (HMSN) typology. This typology demonstrates that the use of figurative language like metaphors can reflect the hybridity of this genre. Droog et al. (2020) showed that metaphorical jokes in satirical news can consist of a combination of one or more of satire’s core communicative functions (e.g., humoristic, informative and evaluative). However, the underlying principles through which metaphors are capable of humorously explaining and/or criticizing current affairs are less clear.

To broaden our understanding of the underlying principles through which metaphorical humor is used in satirical news to fulfill these communicative functions, we integrate the HMSN typology (Droog et al., 2020) with the theoretical framework of the General Theory of Verbal Humor (GTVH; Attardo & Raskin, 1991). The GTVH assumes that verbal humor must draw from six interdependent parameters (also known as Knowledge Resources; Attardo & Raskin, 1991). By combining these two theoretical perspectives (the HMSN typology and the GTVH), we investigate if and how these Knowledge Resources are used to fulfill satire’s core communicative functions, which gives us a better understanding of the characteristics of metaphorical humor, across and between various metaphorical sub-types. With this study we provide more insight into the lower-level discourse features that contribute to the manifestation of satirical news’ hybridity.

## Knowledge Resources of the GTVH as discursive steps in the HMSN typology

**Figure 1.**
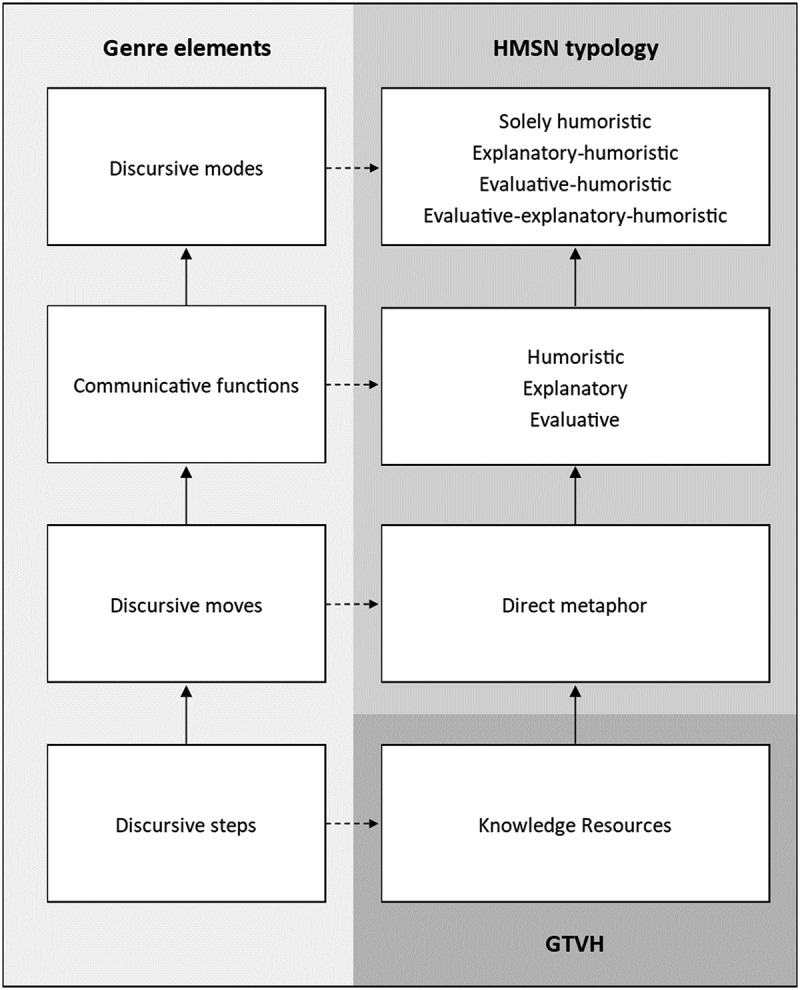
Integration of the HMSN typology and the GTVH.

Note: Paths with continuous lines represent the hierarchy of the genre elements and paths with dashed lines represent the specific form of the genre element as used in the paper. HMSN typology = Humoristic Metaphors in Satirical News typology (Droog et al., 2020), GTVH = General Theory of Verbal Humor (Attardo & Raskin, 1991).

Hybrid genres typically manifest through various *discursive modes* (Baym, 2013). Discursive modes are defined as the communicative orientation of a particular genre element (Baym, 2013). This communicative orientation consists of one or more *communicative functions* (e.g., humoristic, informative and evaluative; Baym, 2013; Droog et al., 2020). These communicative functions can be seen as the purpose or the goals of the genre (Swales, 1990). Communicative functions can be realized through the use of various rhetorical tools that are called *discursive moves* (e.g., direct metaphors; Droog et al., 2020), which are the elements of a text that are used to accomplish one or multiple communicative functions of the genre (Swales, 1990). On an even more specific level of analysis, these discursive moves consist of multiple lower level parts called *discursive steps*, which are the ways by which the discursive moves are realized (e.g., the choice for a specific source domain that makes a metaphor potentially humorous; Droog et al., 2020). When combined, the discursive steps help fulfill the communicative function(s) that the discursive move tries to realize (Swales, 1990). Figure 1 illustrates the relationships between these genre elements.

The hybrid genre of satirical news can be characterized as a balancing act between three communicative functions: providing (1) humor, (2) information, and (3) evaluation about political, economic, or social affairs (Caufield, 2008). Satirists can use various discursive moves to fulfill (particular combinations of) these communicative functions. The HMSN typology showed that metaphors can be used as such a discursive move to realize and switch between four discursive modes that consist of various combinations of satire’s core communicative functions: (1) solely humoristic metaphors, (2) explanatory-humoristic metaphors, (3) evaluative-humoristic metaphors, and (4) complex metaphors, which combine all three communicative functions (Droog et al., 2020; see Figure 1). Solely humoristic metaphors are metaphorical jokes about nonpolitical, noneconomic, and nonsocial affairs (Caufield, 2008). Explanatory-humoristic metaphors are characterized by the use of humor to make sense of difficult political, economic or social affairs, by explaining the unknown in terms of the known (Otieno et al., 2016). Evaluative-humoristic metaphors refer to the use of humor to add (mostly negative) evaluative meaning to political, economic, or social issues affairs (Whaley & Holloway, 1997). Finally, complex metaphors are characterized by the use of humor to both explain as well as criticize political, economic, or social affairs (Droog et al., 2020). However, it is not yet known which specific discursive steps comprise these various metaphorical sub-types, and how these discursive steps contribute to the realization of satire’s core communicative functions.

We argue that, in relation to metaphorical jokes, discursive steps can be operationalized in terms of the Knowledge Resources (KRs) of the GTVH (see Figure 1). According to the GTVH, humorous descriptions are constructed based on six different KRs: (1) script-opposition, (2) logical mechanism, (3) situation, (4) target, (5) narrative strategy, and (6) language (Attardo & Raskin, 1991). Script-opposition (SO) reflects the idea that all instances of verbal humor involve some degree of incongruity (Attardo & Raskin, 1991). To comprehend humor, viewers have to detect and resolve conflict between two semantic scripts, such as two incongruent concepts or situations (e.g., good vs. bad or animal vs. human), that are related in a surprising or unexpected manner. The resolution of that incongruity causes something to be experienced as funny (Attardo & Raskin, 1991). Thus, the GTVH posits that, for humor to work, the two scripts (a) have to overlap in some way and (b) should be opposed to each other (Attardo & Raskin, 1991). Logical mechanism (LM) refers to the (cognitive) mechanisms that connect the different scripts in a humorous description (e.g., a pun, exaggeration, or analogy). Situation (SI) is defined as the overall macro script that describes the background in which the events of the humorous description take place. The target (TA) refers to the actor(s) who become(s) the butt of the joke. Narrative strategy (NS) refers to the way the humorous description is organized in terms of the placement of humor. Finally, language (LA) refers to all the information necessary for the verbalization of the humorous description (Attardo, 2017).

Satirists can use various discursive moves to signal humorous incongruity (i.e., to fulfill the humorous communicative function of satirical news), one of which is the use of direct metaphors (Simpson, 2003). Direct metaphors are cross-domain mappings, in which information from a source domain is explicitly mapped onto a target domain (Lakoff & Johnson, 1980),^1^^1^Metaphors can be divided into two different types: indirect and direct metaphors. For indirect metaphors such as “Donald Trump attacked Hillary Clinton during the debate,” the cross-domain mapping is implied rather than explicitly expressed in language. For direct metaphors such as “This election is like The Hunger Games,” the opposite is true; the cross-domain mapping is explicitly expressed in language (Reijnierse et al., 2018). for example:

(1) *“[Indian] Politics is like a Jalebi* [a spiral shaped sweet snack], *round, round, round, you don’t know where it ends, or where it starts.”* (Patriot Act with Hasan Minhaj, June 2, 2019)

The linguistic structure of this direct metaphor (i.e., A is like B) lends itself well for expressing satirical incongruity humor, because it contains all discursive steps (i.e., KRs) of the GTVH (Attardo & Raskin, 1991; Veale, 2013). Script-opposition is reflected in example (1) in the comparison between the target domain of politics and the source domain of food. The metaphor flag in this example (“is like”) functions as a logical mechanism, by signaling the individual to process the information in the two domains as analogical, which emphasizes the similarities between Indian politics and a Jalebi. The situation is the Indian General Election of 2019 which provides the overall macro script that describes the background in which the events of the metaphorical joke take place, while Indian politics is the entity that is made fun of and serves as the target. With regard to the organization of the placement of humor (narrative strategy), the incongruity between the target (Indian politics) and the source (Jalebi) is not directly resolved by the analogical “A is like B” structure (i.e., the set-up). The information after this structure (*“round, round, round, you don’t know where it ends or where it starts”*) functions as the punchline and is needed to resolve the incongruity between the target and the source, and explains why Indian politics is like a Jalebi. Finally, language is manifested in this example through the use of figurative language (metaphor) to verbalize the information of the joke.

Each metaphorical joke can use the same KRs in different ways. While example (1) compared Indian politics (politics) to a Jalebi (food), other metaphorical jokes might for example compare a politician (politics) to a child (childhood) or to a Sesame Street character (popular culture). In this way, different metaphorical jokes can contain different targets (TA), that relate to different real world situations (SI), that are being joked about through different script-oppositions (SO), with different narrative strategies to do so (NS). This raises the question if the different forms these KRs can take differs across and between the different metaphorical subtypes of the HMSN typology, and thus, if these KRs are used differently to humorously explain and/or criticize current affairs. By analyzing the content of the different metaphorical-subtypes based on the KRs of the GTVH we can analyze the degree of similarity across and between these types of metaphorical jokes. This gives us more insight into the general characteristics of metaphorical humor, and on which characteristics these metaphorical subtypes might differ, and therefore we ask:

*RQ1a: How are the KRs of the GTVH used across the different metaphorical sub-types of the HMSN typology?*

*RQ1b: To what extent are the KRs of the GTVH used differently between the different metaphorical sub-types of the HMSN typology?*

However, to contextualize the similarities and/or the differences in the use of these KRs across and between the different metaphorical sub-types, we need more understanding of how these KRs can work as discursive steps that contribute to the realization of (particular combinations of) satire’s core communicative functions, and therefore we ask:

*RQ2: How are the KRs utilized as discursive steps (a) across and (b) between the different metaphorical sub-types of the HMSN typology?*

Together, answering these research questions can lead to a better understanding of the lower level discourse features that define the hybridity of the genre of satirical news.

## Method

### Materials

To answer our RQs, we collected transcripts of fragments from American satirical news shows from one year (2019). We included nine shows that use satirical humor to criticize current political, economic, or social affairs. Because we wanted to investigate how satirists use metaphors to humorously frame political, economic, and/or societal issues, we only selected satirical monologues. This means that episodes from some satirical news shows were included completely, while of other shows only certain segments were included (see Table 1).

**Table 1.** Satirical news shows and word count included in the corpus.

Satirical news show	Word count	Direct metaphor	Non-humoristic	Solely humoristic	Explanatory-humoristic	Evaluative- humoristic	Complex
Full Frontal with Samantha Bee (monologues)	49,904	80	3	0	0	54	23
Last Week Tonight with John Oliver (full episodes)	48,575	81	6	3	1	57	14
Late Night with Seth Meyers (“a Closer Look” and “the Check In”)	49,661	143	3	0	0	131	9
Patriot Act with Hasan Minhaj (full episodes)	49,844	113	3	2	4	73	31
Real Time with Bill Maher (monologues)	50,305	112	1	0	0	79	32
Saturday Night Live (weekend update)	37,267	74	1	3	0	59	11
The Daily Show with Trevor Noah (monologues)	49,847	104	3	1	1	75	24
The Jim Jeffries Show (monologues)	39,679	31	1	1	1	15	13
The Late Show with Stephen Colbert (monologues)	49,499	63	0	5	0	50	8
Total	424,581	801	21	15	7	593	165

Note: Direct metaphor represents the total of the use of all four metaphorical sub-types of the HMSN typology plus the use of non-humoristic direct metaphors.

The transcripts of these satirical news show segments were collected through the command-line program *youtube-dl* (available at: http://ytdl-org.github.io/youtube-dl/). We randomly chose fragments of these nine shows until we reached a word count of around 50,000 words per satirical news show^2^^2^For *The Jim Jeffries Show* and *Saturday Night Live* there were not enough monologues to reach the 50,000 word count (see Table 1). The total corpus of all the satirical transcripts comprised 424,581 words.

### Coding procedure

#### Direct Metaphors

The satirical transcripts were searched for direct metaphors in two different ways. First, we examined the transcripts with *SketchEngine* (available at: https://www.sketchengine.eu/). We automatically searched the transcripts for metaphor flags (words that can indicate the presence of a marked direct metaphor; Steen et al., 2010a). Examples of metaphor flags are: as (if), like, (so)called, some sort of, in a way or kind of. This search resulted in 5,142 utterances containing one of these words. These utterances were manually read to determine if they contained a direct metaphor of not, based on the steps of the MIPVU (Steen et al., 2010a). To be able to code an utterance as a direct metaphor, the target of the utterance should contrast with its source and should be able to be understood by making a comparison with it. If the source meaning of the utterance is different enough from the target meaning of the utterance (e.g., politics and a jalebi belong to different conceptual domains), and if the two meanings can be related by some form of similarity (they are both, metaphorically speaking for politics and literally speaking for a jalebi, round and you do not know where they end, or where they start) the utterance is coded as a direct metaphor (for more details on this procedure see the full codebook in Online Appendix A at https://osf.io/bk3ra/). Second, since not all direct metaphors contain a metaphor flag, every transcript was also manually read to search the texts for unmarked direct metaphors (which are indicated by the direct expression of their source domain in the text; Steen et al., 2010a). After a trial round on 10% of the data, a second annotator analyzed 20% of the data according to the same coding procedure. Reliability analysis was conducted to examine the extent of agreement between the two annotators. For the direct metaphor coding, a Cohen’s kappa of .80 (indicating substantial agreement; Landis & Koch, 1977) was achieved. Out of the 5,142 utterances containing a metaphor flag, 674 were metaphorical. In addition, we also found 127 unmarked direct metaphors in the satirical transcripts, which resulted in a total of 801 direct metaphors (see Table 1).

#### Communicative functions

All direct metaphors were manually coded for their communicative function(s) (see Online Appendix A for the full codebook, and Online Appendix B for linguistic examples of these communicative functions).

##### Humoristic

Direct metaphors were coded as potentially humorous if the direct metaphor contained (a) a script-opposition, (b) the two scripts overlapped in some way, and (c) if there was only a partial resolution of the incongruity between the target and the source domain (Cohen’s kappa = .66; substantial agreement).

##### Evaluative

Direct metaphors were coded as evaluative if the direct metaphor contained an (indirect) evaluation about the target by criticizing, attacking, or ridiculing them (Cohen’s kappa = .74; substantial agreement).

##### Explanatory

Direct metaphors were coded as explanatory if the direct metaphor tried to increase the listener’s understanding of the target by explaining (something) about the target, or if the direct metaphor tried to explain why the target was evaluated negatively, in the case of evaluative direct metaphors (Cohen’s kappa = .66; substantial agreement).

##### HMSN typology

Direct metaphors containing particular combinations of communicative functions (discursive modes) were clustered into the four metaphorical sub-types of the HMSN typology (Droog et al., 2020). Of the 801 direct metaphors in the corpus, 780 had a humoristic communicative function. The other 21 were non-humoristic direct metaphors with an explanatory and/or evaluative function. Of the 780 metaphorical jokes, 15 were solely humoristic, only 7 were explanatory-humoristic, 593 were evaluative-humoristic, and 165 were humoristic, explanatory and evaluative (i.e., complex metaphors; see Table 1). Because satirists hardly used solely-humoristic and explanatory-humoristic metaphors, we could not run reliable quantitative analyses including these categories (Yates et al., 1999), which is why we excluded these two metaphorical sub-types from the analyses.

#### General Theory of Verbal Humor

Direct metaphors were manually coded for the use of the KRs of the GTVH (Attardo & Raskin, 1991). One of the main criticisms on the GTVH is that various KRs are not fully or adequately operationalized (e.g., Oring, 2011; Ritchie, 2004). To be able to reliably apply the framework of the GTVH onto the large-scale analysis of the use of metaphorical humor in satirical news, we operationalized the KRs of the GTVH based on various studies on humor and figurative language (Attardo, 2017; Curtis & Reigeluth, 1984; Lakoff & Johnson, 1980; Piata, 2016; Veale, 2013). Moreover, because we focused on one discursive move in particular (direct metaphors), two KRs do not vary throughout our analyses. The logical mechanism (LM) KR of all direct metaphors is characterized by the use of analogy, because a direct metaphor consists of an analogical mechanism that connects the target and the source of the metaphor to each other. The language (LA) KR of all direct metaphors is characterized by the use of figurative language, because the use of figurative language is responsible for the precise wording of the joke. Therefore, our analysis focused in detail on the KRs that can take various forms in direct metaphors, which are: script-opposition (SO), situation (SI), target (TA) and narrative strategy (NS; see Online Appendix B for linguistic examples of the various forms of these KRs).

##### Script-opposition (SO)

Script oppositions can be operationalized as conceptual metaphors (Lakoff & Johnson, 1980; Piata, 2016; Veale, 2013). Targets in direct metaphors like “Donald Trump” or “the White House” cluster in larger conceptual structures of politics, while sources in direct metaphors like “fighting” and “attacking” cluster in larger conceptual structures of war. A script-opposition thus represents the mapping between the metaphor’s conceptual source and target domains (Lakoff & Johnson, 1980). Script-oppositions were coded by clustering the targets and sources of the metaphorical jokes into larger conceptual domains. This was done through a bottom-up procedure, in which conceptual domains were created through an iterative process of reading and re-reading all the targets and sources. For each direct metaphor, a potential target and source domain was proposed based on (1) coders’ judgments and (2) the surrounding context of the direct metaphor (Zeng et al., 2021). Next, these proposed domains were compared to the conceptual domains identified in the Master Metaphor List (available through MetaNet at https://metanet.icsi.berkeley.edu/metanet/). If the proposed conceptual domain was not in the list, a new domain was created and its value and necessity as a domain was established through reading and re-reading these metaphorical jokes and their context. The targets of the metaphorical jokes were grouped into 20 different domains. The clustering of the sources required more domains. Since various sources consisted of a blend of multiple domains, it was not possible for some sources to be grouped into only one domain. Ultimately, the sources were clustered into one or more of 42 domains (see online Appendix B for an overview).

##### Situation (SI)

In satirical news, metaphorical jokes always have a referent in the real world (satirists often joke about real politicians, their actions, or their policies). This real world reference is the situation of these metaphorical jokes, because this overall macro script provides the background information individuals need to be able to comprehend the metaphorical joke (Attardo, 2017). Situation was coded by critically reading the linguistic context around the metaphor flag to determine why the target of the metaphor is joked about. The situations were later clustered depending on their (geographical) focus (American, foreign, or both).

##### Target (TA)

The target in a metaphorical joke represents the actor in the “A” part of the “A is like B” structure of the direct metaphor. According to Attardo (2017), targets in humor are generally human or relate to human activity (institutions, practices, beliefs). Satire targets were therefore coded into one or more of 22 categories mostly related to human activity (based on Brugman et al., 2022). The reliability of the categories varied somewhat, but, for most categories, substantial agreement between the two coders was achieved (see online Appendix C for details). Some of the relatively lower kappa scores are a due to the skewness in some of the data. If a specific satire target category is for example only used three times and the coders would disagree on one of these three instances, Cohen’s kappa may be estimated very low (also known as the paradox of kappa; Cicchetti & Feinstein, 1990; Feinstein & Cicchetti, 1990). This is why – next to the kappa scores – we also report percentage agreement. Moreover, due to the large number of satire target categories and the fact that some of these satire target categories hardly occurred, we could not run reliable analyses including all 22 satire target categories. To simplify the analyses, we chose to further cluster these satire targets into one of five larger abstract categories based on the commonalities between them, namely: political individuals, political institutions, organizations, celebrities, and issues (see online Appendix C).

##### Narrative strategy (NS)

The placement of the humorous elements (the set-up and the punchline) in the structure of metaphorical jokes can differ in three different ways. In the simple narrative strategy (this NS is inspired by the classification of analogies in Curtis & Reigeluth, 1984), there is no clear distinction between the set-up of the metaphorical joke and the punchline. In this case, the punchline is already directly a part of the “A is like B” set-up. This means that the incongruity between the target and the source is directly resolved by the “A is like B” structure. The grounds on which the comparison between the target and the source are based are not explicitly stated and are left to the audience for interpretation.

In the enriched narrative strategy (this NS is inspired by the classification of analogies in Curtis & Reigeluth, 1984), the setup of the humorous metaphor (A is like B), and the punchline of the metaphor (i.e., the explanation of why A is like B) are separate. In this case, the incongruity between the target and the source is not resolved by the “A is like B” structure (i.e., set-up), but the sentence after this structure is needed to resolve the incongruity (i.e., the punchline).

The extended narrative strategy (this NS is inspired by the classification of analogies in Curtis & Reigeluth, 1984) is a combination of the previous two structures. In this case, the punchline is again already directly part of the “A is like B” set-up, which means that the incongruity between the target and the source is resolved by the “A is like B” structure. However, in this narrative strategy, there is some extra information after this structure that adds a little bit of extra humor to the joke, but is not needed to resolve the incongruity between the target and the source (Cohen’s kappa =. 80; substantial agreement).

## Results

### Occurrence of knowledge resources in the HMSN typology

Table 2 gives an overview of the occurrence of the KRs across and between the metaphorical subtypes. In relation to *RQ1a*, the results show little variation in the use of the KRs across the metaphorical sub-types. First of all, almost 70% of the metaphorical jokes contained a politics target domain, and although the sources of the metaphorical jokes could be clustered into one or more of 42 conceptual domains, as many as 28% of the metaphorical jokes contained a popular culture source domain. Therefore, the most frequent scrip-opposition was politics-popular culture, which comprised 21% of the corpus. Furthermore, in general, American satirical news shows have a strong inward focus, in that 84% of all the metaphorical jokes revolved around American rather than international situations. Moreover, most of the metaphorical jokes (58%) contained a person-oriented satire target (TA), while only 29% of the metaphorical jokes contained an issue-oriented satire target (TA). Finally, the simple narrative strategy was used in more than half of all the metaphorical jokes (56%).

**Table 2.** Relation between Knowledge Resources and metaphorical jokes in the HMSN typology.

Knowledge resources	Evaluative-humoristic	Complex metaphors	Total
**Script-opposition**			
***Target domain***			
Politics	426	112	538
Money	33^a^	21^b^	55
Popular culture	26	5	31
News	25	3	28
Other	83	24	107
***Source domain***			
Popular culture	176	40	216
Sex	40	18	58
Objects	48	7	55
Food	44	10	54
Other	407	115	522
**Situation**			
American	502	136	638
Foreign	49	14	63
Both	42	15	57
**Target category**			
Political actors	313^b^	52^a^	365
Political organizations	46^a^	27^b^	73
Organizations	16^a^	10^b^	26
Celebrities	64^b^	8^a^	72
Issues	154^a^	68^b^	222
**Narrative strategy**			
Simple	356^b^	71^a^	427
Enriched	66^a^	57^b^	123
Extended	171	37	208

Note: Superscript “a” indicates that a particular category of a KR was used relatively less often than expected in a metaphorical sub-type, while superscript “b” indicates that a particular category of a KR was used relatively more often than expected in a metaphorical sub-type.

To investigate if and how some of these KRs were used differently across the different metaphorical sub-types (*RQ1b*), we conducted various chi-square analyses. These chi-square analyses show no differences in the use of script-oppositions (SO)^3^^3^To make the chi-square analyses of the target and source domains (script-opposition) more comprehensible, we only used the four most common target and source domains and grouped all other domains into the category other. Our results show that the use of target domains did differ by the type of metaphorical jokes (χ2(4) = 12.20, *p* = .016; Cramer’s *V* = .13). However, based on the adjusted standardized residuals we see that only the target domain of money was used in complex metaphorical jokes relatively more often than expected and relatively less often in evaluative metaphorical jokes than expected. Therefore, we argue that there are almost no differences in the use of these target domains across the various types of metaphorical jokes. Because the sources could be grouped into multiple source domains, we conducted a chi-square analysis for each domain separately. These results indicate that the use of source domains did not differ by the type of metaphorical jokes: popular culture (χ2(1) = 1.87, *p* = .171; Cramer’s *V*;= .05); sex (χ2(1) = 3.17, *p* = .075; Cramer’s *V* = .07); food (χ2(1) = 0.36, *p* = .548; Cramer’s *V* = .02); objects (χ2(1) = 2.87, *p* = .092; Cramer’s *V* = .06); other (χ2(1) = 0.68, *p* = .794; Cramer’s *V* = .01). and situations (SI) (χ2(2) = 0.78, *p* = .678; Cramer’s *V* = .03) between the metaphorical sub-types. In contrast, the use of satire target categories (TA) (χ2(4) = 41.35, *p* < .001; Cramer’s *V* = .23) and narrative strategies (NS) (χ2(2) = 52.18, *p* < .001; Cramer’s *V* = .26) did differ between the metaphorical sub-types. The adjusted standardized residuals indicate that satire target categories such as (political) organizations and issues, and the enriched narrative strategy were used relatively more often than expected in complex metaphorical jokes and relatively less often than expected in evaluative metaphorical jokes, while satire target categories such as political actors and celebrities, and the simple narrative strategy were used relatively less often than expected in complex metaphorical jokes and relatively more often than expected in evaluative metaphorical jokes.

### Knowledge Resources as discursive steps in the HMSN typology

Our second research question considered how the KRs are utilized as discursive steps (a) across and (b) between the different metaphorical sub-types of the HMSN typology.

#### The use of Knowledge Resources across the metaphorical sub-types

Although we found no variation in the use of the script-opposition KR between the different metaphorical sub-types, selecting a particular humorous target-source domain combination over others can still be utilized as a discursive step to help fulfill the explanatory and/or evaluative functions of satirical news. Direct metaphorical jokes have communicative power and can work as perspective changers, by providing a new perspective onto the target domain of the joke by explicitly drawing attention to the source domain of the metaphorical joke (Simpson, 2003; Steen, 2016). In this way, script-oppositions can be used as a discursive step to highlight and hide, by invoking a certain new perspective of the target over others, which is deemed most appropriate for the communicative goals of the metaphorical joke. For example:

(2) *“After he met with Trump, Lev Parnas told two of his buddies he was on ‘a secret mission’ to pressure the Ukrainian government to investigate Joe Biden, like some sort of James Bond mission. Yes, a James Bond mission. The one thing you don’t want in a spy is someone excited to tell everyone they’re a spy: ‘My name’s James Bond, and I’m a spy! Here’s my license to kill. Well, it’s a learner’s permit right now. Don’t look at the picture. I have a tuxedo on under my wet suit, and it’s really binding in the crotch!’”*

(The Late Show with Stephen Colbert, November 22, 2019)

Example (2) contains a politics (Lev Parnas was one of the key figures in the Trump-Ukraine controversy) vs. popular culture (James Bond) script-opposition. In this example, Stephen Colbert criticizes Lev Parnas by comparing his actions to the actions of a fictional spy. This example is different from Example (1), where emphasis was placed on the similarities between the target (Indian Politics) and the source (Jalebi). Example (2) however, emphasizes the differences between the source and the target, by highlighting the incongruity between them by using an ironic rebuttal (Colston & Gibbs, 1998). James Bond is typically seen as a spy who can solve the most difficult missions, while Colbert describes the target as someone who makes certain basic mistakes as a spy. Colbert uses this ironic rebuttal analogy to explain how the target, Lev Parnas, is actually not like the source, James Bond (Whaley & Holloway, 1997). The use of this ironic rebuttal analogy indicates that, for metaphors, highlighting the dissimilarities between the target and the source domains (instead of focusing on the similarities) can also be used as a discursive step to express criticism (Steen, 2016; Whaley & Holloway, 1997).

Besides, some (political) situations may be worthier of satirization than others. According to Holbert (2016), the likelihood of a situation becoming the focus of a satirical attack depends on the perceived threat (to democracy) and the severity of the situation. The greater a situation is perceived as a (severe) threat (e.g., a politician lying under oath vs. a politician making a gaffe in a public performance), the likelier that the satirical criticism will become harsher (Holbert, 2016). This harshness can manifest in the particular target-source domain combination that the metaphorical joke contains, in that some combinations of target situations with particular source domains result in harsher metaphorical jokes than others (Droog et al., 2020). When satirists for example want to criticize the behavior of a politician who lied under oath, they can choose a source domain that in comparison with the target situation is perceived as (a) less severe (e.g., comparing the politician to a child or a bratty teen; which results in mild criticism), as (2) evenly severe (e.g., comparing the politician to a celebrity who did something illegal; which results in moderate criticism), or (3) as even more severe (e.g., comparing the politician to a dictator; which results in harsh criticism). This means that the discursive steps regarding the situation KR thus may not lie in the selection of a specific situation for the metaphor, but in the selection of a particular script-opposition that is deemed appropriate for the particular situation (Droog et al., 2020; Holbert, 2016).

#### The use of Knowledge Resources between the metaphorical sub-types

The previous quantitative analysis showed some variation in the use of the target (TA) KR between the metaphorical sub-types. These results can possibly be explained by the level of abstraction of these satire target categories. Abstraction consists of two dimensions: concreteness and precision (Iliev & Axelrod, 2017). Concreteness is related to physicality, in that physical entities that can be seen, touched, or heard (e.g., a pencil) are considered as more concrete than nonphysical entities (e.g., emotions). Precision is related to specificity, in that the more information that is provided about an entity, the more precise it is (Iliev & Axelrod, 2017). We argue that the different satire target categories that are used in the metaphorical jokes can differ on these dimensions, which makes some satire target categories more appropriate for certain metaphorical subtypes than other satire target categories.

To illustrate, political or societal issues (e.g., the voting system or student loans) and (political) organizations (e.g., the World Trade Organization or Amazon) are more abstract because they are nonphysical and quite unspecific, since they refer to a whole issue or organization. Political actors or celebrities on the other hand (e.g., Donald Trump or Jeff Bezos) are more concrete because they are physical entities and are quite specific, since they involve only one person (Iliev & Axelrod, 2017). Abstract satire targets, such as political or societal issues or (political) organizations, probably require more explanation than more concrete satire targets, which makes these abstract satire targets more likely to appear in complex metaphors and less likely to appear in evaluative-humoristic metaphors. The opposite is probably true for concrete satire targets such as political actors or celebrities. Because of the physicality and preciseness of these satire targets, they probably do not require more explanation in comparison with more abstract satire target. This makes these concrete satire targets less likely to appear in complex metaphors and more likely to appear in evaluative-humoristic metaphors (Iliev & Axelrod, 2017). This means that this target KR may constrain the selection of a particular metaphorical sub-type, in that often a particular metaphorical-subtype is selected with a communicative function that best suits the nature (i.e., abstraction) of the satire target that is being joked about.

In turn, the use of a certain metaphorical sub-type that is most appropriate for the abstraction of the satire target of the metaphorical joke, can also help explain the variation we found in the use of the narrative strategy KR between the metaphorical sub-types. This variation is probably related to the differences in the structures of the narrative strategies, in that some structures are more suitable to help fulfill certain kind of communicative functions than other structures. The structures of the simple and the enriched narrative strategy differ in the addition of some form of causation, in that a simple narrative structure is more descriptive whereas an enriched narrative structure is more explanatory (Curtis & Reigeluth, 1984; Harrison & Treagust, 2006).

This difference can explain why the simple narrative strategy is used relatively less often in complex metaphorical jokes and more often in evaluative metaphorical jokes: since concrete satire targets are used relatively more often in evaluative metaphorical jokes, the use of a more descriptive and less information rich structure, like the simple narrative strategy, to criticize the satire target, is probably sufficient enough to be able to understand the meaning metaphor (Iliev & Axelrod, 2017). To illustrate:

(3) *“Democrats snipe and bitch at each other – they’re like that couple that’s divorcing but came to the dinner party anyway.”*

(Real Time with Bill Maher, May 31, 2019)

The simple narrative strategy is a suitable structure for metaphorical jokes where the relationship between the target and the source is highly obvious and need little to no explanation (Curtis & Reigeluth, 1984). This is also the case in this example (3), where the satirist explicitly describes his view on the behavior of the satire target category. Therefore, the satirist does not have to explain why he believes that the behavior of the Democrats is like the behavior of a divorcing couple. This structure allows the satirist to provide a descriptive evaluation about the satire target of the metaphorical joke, and is used to argue that the Democrats are constantly fighting with each other. Although this simple narrative strategy is sometimes used as the structure of complex metaphorical jokes as well, this strategy is particularly useful for satirists as a discursive step to criticize current affairs.

Moreover, because satirists relatively more often use complex than evaluative-humoristic metaphors to talk about abstract topics, the satirists probably also (have to) use the enriched narrative structure relatively more often, since they need to give a more precise, specific and detailed explanation of why the target is like the source, to clarify the abstract satire target (Iliev & Axelrod, 2017). This can explain why the enriched narrative strategy is used relatively more often in complex metaphorical jokes and relatively less often in evaluative metaphorical jokes, because the structure of the enriched narrative strategy lends itself well for explanations. To illustrate:

(4) *“The Middle East. It’s like the New York Knicks. You know? It’s got major problems, and it’ll probably be generations before they’re fixed.”*

(The Daily Show with Trevor Noah, September 18, 2019)

In this example (4), Trevor Noah compares the complex, abstract and tense situation in the Middle East (e.g., the U.S. war in Afghanistan) with the situation of the NBA team New York Knicks (e.g., bad reputation, poor sporting results, managerial and coaching issues). The specific structure of this metaphorical joke allows the satirists to precisely explain the exact grounds on which the comparison between the Middle East and the New York Knicks are based. This helps people better understand the complex and abstract situation in the Middle East, by making it more concrete and precise (Iliev & Axelrod, 2017). The fact that the structure of the enriched narrative strategy lends itself well for explanations, does not mean that this structure is not suitable for evaluations. In fact, this metaphorical joke is also used to argue that there are huge problems in the Middle East that are not easily solved. This means that the enriched narrative strategy can be used by satirists in (complex) metaphorical humor as a discursive step to both explain and criticize current affairs.

Finally, the use of the extended narrative strategy did not differ between the different metaphorical sub-types. This is probably because the extended structure lends itself well for both explanation and argumentation (Curtis & Reigeluth, 1984). This structure allows satirists to make more elaborate descriptive evaluations than in the simple structure, but can also be used in an explanatory way, because this structure creates the possibility for extra mappings between the target and the source, which makes the grounds on which the comparison is based more explicit (Curtis & Reigeluth, 1984; Harrison & Treagust, 2006). This means that the extended narrative strategy can be used by satirists in (complex) metaphorical humor as a discursive step to both explain and criticize current affairs, and that the narrative strategy KR in general, can be used as a discursive step to help fulfill a particular communicative function in the metaphorical joke.

## Discussion and conclusion

The aim of this paper was to shed light on the lower-level discourse features that contribute to the manifestation of satirical news’ hybridity, by investigating how and to what extent the Knowledge Resources of the GTVH are used to help fulfill the communicative functions in the different metaphorical sub-types of the HMSN typology.

*RQ1* considered the (differences in) the use of the KRs of the GTVH across and between the different metaphorical sub-types of the HMSN typology. We found that, overall, there is little variation in the use of the KRs across the different metaphorical jokes. In general, metaphorical jokes in satirical news were characterized by the use of a politics-popular culture script-opposition (SO), an American situational focus (SI), a person-oriented satire target (TA), and a simple narrative strategy (NS). However, we did find that the use of some of the KRs differed between the metaphorical subtypes. Although there were no differences in the use of the script-opposition (SO) and the situation (SI) KRs, we found that complex metaphors were characterized by the use of relatively more satire target categories (TA) such as (political) organizations and issues and by an enriched narrative strategy (NS), while they contained relatively fewer political actors and celebrities as satire targets (TA) and a simple narrative strategy (NS) as the structure of the metaphorical jokes. Evaluative metaphors were characterized by the use of relatively more satire target categories such as political actors and celebrities (TA), and by a simple narrative strategy (NS), while they contained relatively fewer (political) organizations and issues as satire targets (TA) and an enriched narrative strategy as the structure of the metaphorical jokes (NS).

These results build on the HMSN typology because it is the first study that empirically validated this theoretical model, by investigating to what extent the metaphorical sub-types of this typology are used in satirical news (Droog et al., 2020). Our findings show that not all metaphorical sub-types of the HMSN manifest evenly throughout the genre of satirical news. Direct metaphors in satirical news are typically used to reflect a humorous-evaluative discursive mode, and are sometimes completed with an explanatory function, when the satire target requires an explanation. This means that the explanatory function of satirical news is secondary to its humorous and evaluative functions. These results support perspectives that propose that satirical news is a form of humorous discourse wherein satire is primarily seen as a discursive practice amongst a target, a satirist and an audience with the purpose of criticizing or ridiculing the target, resulting in humor between the satirist and the audience (Simpson, 2003). However, based on the findings of this study, differences in the use of explanatory metaphors between different satirical news shows could be expected. Since explanatory metaphors are more often used when discussing abstract satire targets, satirical news shows that are known for their in-depth analysis of relatively unknown abstract issues (such as *Last Week Tonight with John Oliver*) might use more explanatory metaphors in their satirical monologues, than satirical news shows that contain shorter and less substantive satirical monologues (e.g., *Saturday Night Live*).

With *RQ2*, we contextualized the similarities and differences in the use of these KRs by asking how these KRs were used as discursive steps across and between the different metaphorical-subtypes. Here, we observed a number of differences in the ways the KRs function in metaphorical humor. First of all, we found that certain KRs can work as discursive steps to help fulfill the communicative function(s) of a metaphorical joke. For example, choosing a particular target-source domain combination (SO) over others can be used as a discursive step to highlight and hide, by invoking a certain new perspective of the target over others, which is deemed most appropriate for the communicative goals of the metaphorical joke. Moreover the use of a particular narrative strategy (NS) can also help fulfill the communicative goals of a metaphorical joke, in that the structure of the simple narrative strategy is more appropriate for humoristic evaluations, while the enriched narrative strategy lends itself well for humoristic evaluative explanations. Second, certain KRs seem to constrain the different forms that the metaphorical jokes can take, in that the selection of a particular satire target (TA) of a metaphorical joke can constrain the selection of a particular metaphorical sub-type, in that often a particular metaphorical-subtype is used with a communicative function that best suits the nature (i.e., abstraction) of the satire target that is being joked about. In addition the KRs seem to constrain the options available for the other KRs, in that for example some script-oppositions are more appropriate for the severity of a specific situation (SI) in a metaphorical joke than others. However, the severity of these issues and the harshness of their accompanying script-oppositions (Droog et al., 2020) were not coded in the current analysis. Therefore, to be able to better understand why satirists use certain script-oppositions over others during particular situations, future research should strive to further operationalize these concepts, so that they can reliable be applied onto (large) data sets (Droog et al., 2020).

These results build on the GTVH because our findings suggest that the traditional hierarchical order of the KRs may not be entirely applicable to the genre of satirical news (Attardo & Raskin, 1991). Attardo and Raskin (1991) argue that the KRs are hierarchically organized and have an interdependent relationship with each other (the order is: script-opposition; logical mechanism; situation; target; narrative strategy; language). This means that the higher level KRs will determine the lower level KRs by limiting the number of forms they can take (e.g., certain script-oppositions can only work through certain narrative strategies). In relation to metaphorical humor in satirical news, we suggest that the situation rather than the script-opposition is the highest order KR. This is because the goal of satirical news is to provide humorous explanations and/or criticism on real world current affairs. Therefore, satirical jokes start from certain situations in the real world that are worthy of satirization. This in turn limits the options available for the satire target of the metaphorical joke, because often only certain actors are involved in these real world situations. Consequently, certain discursive modes fit better with the abstractness of the satire target of the metaphorical joke, which in combination with the severity of the situation, might constrain the selection of an appropriate script-opposition. Finally, all of these previous KRs might limit the options available for a particular narrative strategy that best suits the communicative goals of the metaphorical joke. The original hierarchal order of the KRs as proposed by the GTVH has been previously challenged and criticized (e.g., Oring, 2011). Even the authors of the GTVH itself were not able to fully verify this hierarchal order. Ruch et al., (1993) conducted an experiment in efforts trying to confirm the hierarchy of the KRs of the GTVH. While the hierarchy of some lower parts of the GTVH were confirmed, they also found that the situation KR preceded the script-opposition KR, which is also in line with the findings of our analysis. Future research may corroborate our hypothesis by conducting interviews with the scriptwriters of satirical news shows.

In addition, the findings of our study also provide more insights into metaphors, because this is the first study that operationalized and applied the analytical framework of the GTVH onto the large scale analysis of humorous metaphors in satirical news shows. In this way, this study has shown that direct metaphors can be used in different ways to fulfil the communicative functions of satire, which makes the use of these direct metaphors a characterizing discursive move of the hybrid genre of satirical television news. At the same time, the fact that we only investigated the use of such verbal direct metaphors, presents the first caveat of our study. Namely, indirect metaphors, compared to direct metaphors, occur more frequently across genres (Steen et al., 2010b). Although not all KRs of the GTVH can be as easily applied to the structure of indirect metaphors (because the target and the source domain of indirect metaphors are not explicitly expressed in language), research shows that indirect metaphors can, just like direct metaphors, exert communicative power (e.g., Charteris-Black, 2011). Moreover, satirical news shows also use over-the-shoulder graphics that often contain visual metaphors. Although the GTVH is currently not suited to analyze visual humor, future research could strive to examine if and how the framework of GTVH can be applied onto the study of indirect and visual metaphors in satirical news shows. In this way, future scholars can investigate which types of discursive steps comprise the indirect and visual metaphors that are used in satirical news as discursive moves to realize satire’s core communicative functions.

Another caveat of our study is that we only investigated the use of metaphorical jokes in American satirical news outlets. We specifically chose American satire because, compared to many other countries, the US broadcasts a wide variety of satirical news shows. This allowed us to draw conclusions that transcend the level of individual shows. However, there are various reasons to believe that the content of American satire is different than that of for example European satirical news. European (satirical) news for example seems to be more issue-oriented and more internationally focused than American (satirical) news (Matthes & Rauchfleisch, 2013; Wilke et al., 2012). Moreover, researchers have also observed differences in nations’ humor cultures (Baym & Jones, 2012), in that different types of countries also prefer different types of humor. This might also influence the type of metaphorical jokes satirists make in various countries. Therefore future research should seek to replicate this study in cultural contexts different from the USA, to check for the robustness of our findings. In addition, in order the advance our knowledge of the use of (direct) metaphors in satirical news, future research should apply the current analytical framework onto the study of other satirical news outlets as well (e.g., written satirical news articles on websites such as *The Onion*).

In sum, our study showed that satirists mainly use metaphorical humor to criticize or ridicule current affairs and less to explain them. Besides, our study also showed little variation in the use of the KRs across the various types of metaphorical jokes, while we found some variation in the use of the KRs between the different metaphorical sub-types. We contextualized these similarities and differences by showing two different ways in which the KRs function in metaphorical humor: (1) certain KRs function as discursive steps to help fulfill certain communicative functions of satirical news, and (2) certain KRs seem to constrain the different forms that the metaphorical jokes can take, by limiting the selection of a particular metaphorical sub-type, or the available options the other KRs can take. Overall, the integration of the GTVH and the HMSN typology has given us more insight into the lower-level discourse features that contribute to the manifestation of satirical news’ hybridity, by providing a better understanding of the underlying discursive steps through which metaphorical humor is used in satirical news to inform the audience and/or to criticize current affairs.
